# Genital microbiota of women using a 90 day tenofovir or tenofovir and levonorgestrel intravaginal ring in a placebo controlled randomized safety trial in Kenya

**DOI:** 10.1038/s41598-022-13475-9

**Published:** 2022-07-14

**Authors:** Smritee Dabee, Nelly Mugo, Victor Mudhune, Eleanor McLellan-Lemal, Sue Peacock, Siobhan O’Connor, Betty Njoroge, Beatrice Nyagol, Andrea R. Thurman, Eunice Ouma, Renee Ridzon, Jeffrey Wiener, Harald S. Haugen, Melanie Gasper, Colin Feng, Shannon A. Allen, Gustavo F. Doncel, Heather B. Jaspan, Renee Heffron, Kisumu Combined Ring Study Team, Nelly R. Mugo, Victor Mudhune, Renee Heffron, Eleanor McLellan-Lemal, Siobhan O’Connor, Gustavo F. Doncel, Andrea R. Thurman, Betty Njoroge, Beatrice Nyagol, Eunice Ouma, Richard Ndivo, Maxcine Oguta, Haynet Opon, Dorine Awili, Anne Mithika, Epines Chavangi, Jecinter Oruko, John Okanda, Arthur Ogendo, Elizabeth Ayuo, Evans Odipo, Vitalis Sewe, Boaz Kerubo, Calvin Mbeda, Eucabeth Awuonda, Isdorah Odero, Emily Anyango, Erica Mimba, Fredrick Oloo, Richard Odipo, Valarie Opollo, Emily Kerubo, Fredrick Omondi, Angelica Gende, Kelvin Wandera, Carolyne Juma, Alice Were, Phoebe Ogutu, Susan Aremo, Philister Madiega, Daniel Ogando, Judith Arego, Margaret Otieno, Rosemary Akello, Ken Ondeng’e, Teresa Omoro, Caren Amondi, Kimberly McCarthy, Renee Ridzon, Susan Morrison, Meighan Krows, Connie Celum, Abenan L. Ouattara, Nazita Yousefieh, Jill Schwartz, Allison Matthews, Shannon Allen, Elizabeth Russell, Karen Near, Jeffrey Wiener, Taraz Samandari, Stacie R. Deaton, Lee Claypool, Josh E. Betts, Richard E. Haaland, Amy Martin, Jeffrey Fountain, Terry A. Jacot, David Erikson, Steven W. Blue, Heather Jaspan, Smritee Dabee, Colin Feng, Melanie Gasper, Barrett Remington, Bruce L. Frank, Nina Isoherranen, Harald Haugen, Jared Baeten, Katherine Thomas, Athena Kourtis, Naomi Tepper, Lisa Ondrejcek, Angela Williams, Matt Johnson, Joe Jiang, Sue Peacock, Deborah Donnell

**Affiliations:** 1 Seattle Children’s Research Institute, 307 Westlake Ave N, Seattle, WA USA; 2 University of Washington Global Health, 325 Ninth Ave, Box 359927, Seattle, WA USA; 3 Center for Clinical Research, Kenya Medical Research Institute, Nairobi, Kenya; 4 Center for Global Health Research, Kenya Medical Research Institute, Kisumu, Kenya; 5 Division of HIV/AIDS Prevention, Centers for Disease Control and Prevention, Atlanta, GA USA; 6 CONRAD, Eastern Virginia Medical School, Norfolk, VA USA; 7 US National Institutes of Health, Bethesda, MD USA; 8 United States Agency for International Development, Washington, DC USA; 9 Institute of Infectious Diseases and Molecular Medicine, University of Cape Town, Cape Town, South Africa; 10 ICF, Atlanta, GA USA; 11 Endocrine Technologies Core, Oregon National Primate Research Center, Beaverton, OR USA; 12 Particle Sciences, Bethlehem, PA USA; 13 Division of Birth Defects and Infant Disorders, United States Centers for Disease Control and Prevention, Atlanta, GA USA; 14 DF/Net, Inc., Seattle, WA USA; 15 Fred Hutchinson Cancer Research Center, Seattle, WA USA

**Keywords:** Randomized controlled trials, Microbiome

## Abstract

In a phase-IIa trial, we investigated the influence of 90 days continuous-delivery tenofovir (TFV) intravaginal rings (IVRs) with/without levonorgestrel (LNG) on the genital microbiota of Kenyan women. Eligible women (n = 27; 18–34 years; negative for HIV, sexually transmitted infections, and Amsel-bacterial vaginosis) were randomized 2:2:1 to use of IVRs containing TFV, TFV/LNG, or placebo. Using vaginal wall and IVR swabs at IVR insertion and removal, the genital microbial composition was determined using 16S rRNA gene sequencing. The presence of *Candida* spp. was determined using qPCR. The vaginal total bacterial burden appeared to decrease with TFV and TFV/LNG IVR use (log_10_0.57 and log_10_0.27 decrease respectively; p > 0.05). The TFV/LNG IVR was more ‘stabilizing’: 50% of the participants’ microbiota community state types remained unchanged and 50% shifted towards higher *Lactobacillus* abundance. Specifically, TFV/LNG IVR use was accompanied by increased abundances of *Lactobacillus gasseri/hominis/johnsonii/taiwanensis* (16.3-fold) and *L. fermentum/reuteri/vaginalis* (7.0-fold; all p < 0.01). A significant shift in the overall microbial α-diversity or β-diversity was not observed for either IVR, and IVR use did not influence *Candida* spp. prevalence. TFV/LNG and TFV IVRs did not adversely affect the genital microbiota and are safe to use. Our findings support further studies assessing their efficacy in preventing HIV/HSV-2 and unintended pregnancies.

## Introduction

With global estimates of 1.7 million incident HIV infections^1^ and 121 million unintended pregnancies annually^2^, development of a multipurpose, user-controlled product that prevents heterosexual transmission of HIV and provides contraception for women is invaluable. Daily oral emtricitabine (FTC)/tenofovir disoproxil fumarate (TDF) has revolutionized HIV prevention as the first pre-exposure prophylaxis (PrEP) option. However, a high level of adherence with the dosing regimen is essential for protection and is often difficult to achieve^3,4^. Long-acting contraception options not requiring daily adherence have been pivotal in the prevention of unintended pregnancies. Innovative multipurpose technologies (MPT) could be developed by leveraging current long-acting contraceptive strategies with HIV PrEP co-delivery or co-formulation to improve protective coverage.

Intravaginal rings (IVRs) are a safe, discreet and effective option for simultaneous delivery of contraception and antiretrovirals^5^. An IVR with dapivirine, which was found to reduce HIV incidence by 27% in two efficacy trials^6,7^, with up to 75–91% reduction among women with higher adherence to the IVR^8^, received a positive opinion from the European Medicines Agency and the World Health Organization^9,10^. IVRs also allow for consistent contraceptive hormone release, eliminating the high peak experienced with injectables^11^ or the daily fluctuations from oral contraceptives^12^.

Given their localized effects, the influence of IVRs on the genital microbiota is an important consideration. Microbiota shifts could influence genital health and ability of the female genital tract (FGT) to protect against infections including sexually transmitted infections (STIs). In one study of coitally-dosed 1% tenofovir (TFV) vaginal gel (a product that has not moved forward in development)^13^, efficacy was higher in the presence of a *Lactobacillus*-dominant microbiota but decreased in the presence of higher mucosal inflammation^14,15^.

The CONRAD co-formulated TFV/levonorgestrel (LNG) and single agent TFV IVRs have been tested for safety in a phase-I trial among women from the United States (US) and the Dominican Republic^16^. However, the effect of these IVRs on the genital microbiota of sub-Saharan African Black women has not been evaluated. Women in this region face a disproportionate burden of HIV and their genital microbiota has been shown to be markedly different to women with different lived experiences and ethnicities. As part of the primary goals for this phase-IIa safety trial of 90 days use TFV and TFV/LNG IVRs, we determined the effect of each IVR relative to the placebo IVR on the genital microbiota of African women. In addition, we assessed the interplay between IVR use and genital microbiota on local TFV levels and the incidence of candidiasis.

## Results

### Participant characteristics

Twenty-six of the twenty-seven randomized women had bacterial absolute abundance data available: TFV/LNG IVR (n = 11), TFV IVR (n = 10), and placebo IVR (n = 5). The median age was 22 years [interquartile range (IQR) 21–26], median body mass index was 22.4 [IQR 19.8–24.9], and demographic characteristics were similar across study arms (Table 1). Most women used male condoms during the study (74.1%), 7.4% previously used a copper-intrauterine device (≥ 6 months before enrolling in the study), and 18.5% used no contraceptive method. Despite being asymptomatic and BV-negative by Amsel criteria at screening, 4/27 (14.8%) women were Nugent-BV-positive at the time of IVR insertion and 11/27 (41.0%) at the IVR removal visit. With the exception of an estimated increase in the prevalence of BV-positive Nugent score, differences in clinicial characteristics at IVR removal were small.

**Table 1:** Characteristics of women randomized to use 90 days intravaginal rings (IVR) with continuous delivery of tenofovir and levonorgesterel (TFV/LNG), tenofovir (TFV), or placebo, Kisumu Kenya, 2019.

	TFV + LNG n = 11 Median (IQR) or N (%)	TFV n = 11 Median (IQR) or N (%)	Placebo n = 5 Median (IQR) or N (%)
Age	21 [20–24]	25 [22–32]	23 [21–23]
BMI	20.2 [18.4–24.9]	22.9 [21.1–26.2]	23.7 [21.2–24.3]
Menstrual cycle length	31 [26.5–35.0]	28 [26.0–34.0]	31 [30.0–34.5]
**Contraceptive method at enrollment**			
None	2 (18.2%)	2 (18.2%)	1 (20.0%)
Condoms	8 (72.7%)	8 (72.7%)	4 (80%)
Cu-IUD	1 (9.1%)	1 (9.1%)	0 (0.0%)
**Nugent score at IVR insertion**			
0–3 (Negative)	7 (63.6%)	9 (81.2%)	3 (60.0%)
4–6 (Intermediate)	3 (27.3%)	1 (9.1%)	0 (0.0%)
7–10 (Positive)	1 (9.1%)	1 (9.1%)	2 (40.0%)
**Nugent score at IVR removal**			
0–3 (Negative)	6 (54.5%)	3 (27.3%)	2 (40.0%)
4–6 (Intermediate)	1 (9.1%)	3 (27.3%)	1 (20.0%)
7–10 (Positive)	4 (36.4%)	5 (45.4%)	2 (40.0%)
Vaginal pH at baseline	5.2 [4.6–5.5]	4.9 [4.6–5.2]	4.9 [4.9–4.9]
Vaginal pH at ring removal	5.2 [4.9–5.5]	5.2 [4.2–5.5]	4.6 [4.4–5.5]
Time on IVR, days	46 [21–89]	90 [40–91]	68 [67–91]

*IQR* interquartile range, *BMI* Body mass index, *Cu-IUD* Copper intrauterine device.

### Total bacterial load did not change with IVR use

**Figure 1:**
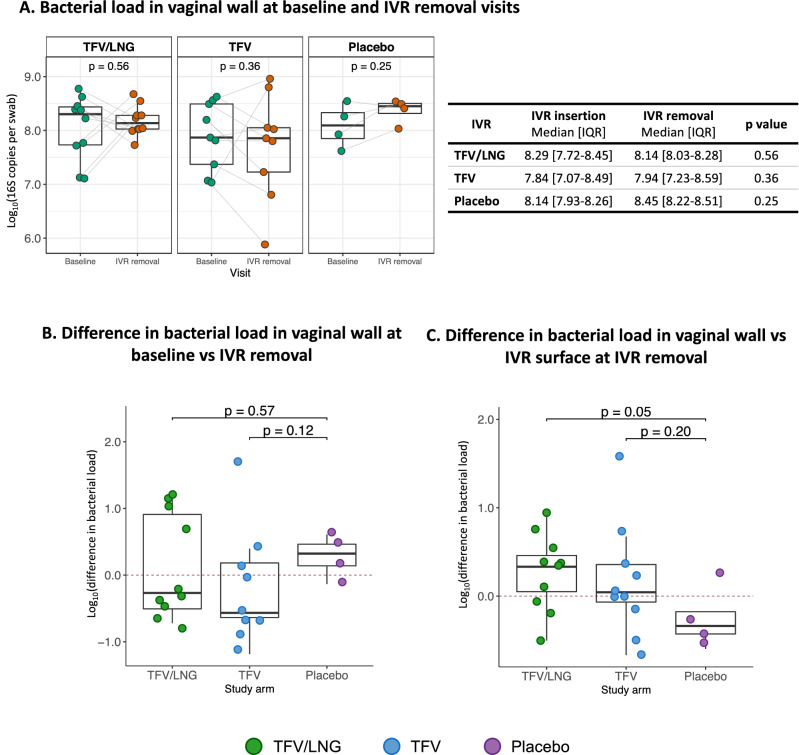
Change in bacterial load between intravaginal ring (IVR) insertion and removal among women randomized to use tenofovir/levonorgestrel (TFV/LNG), tenofovir (TFV), and placebo IVRs, Kisumu, Kenya, 2019. Comparing (**A**) vaginal log_10_ total 16S copies per swab between the two visits, (**B**) the degree of change in vaginal wall log_10_ total bacterial 16S copies from baseline to the IVR removal visit among women and (**C**) difference in total log_10_ 16S copies between the vaginal wall and IVR surface (log_10_ bacterial load of vaginal wall − log_10_ bacterial load of IVR) at the time of IVR removal. Values above and below the dotted red line indicate an increase and decrease in bacterial loads respectively. p values were determined using the Wilcoxon Signed Rank test (**A**) and Mann–Whitney U test (**B,C**).

The total FGT 16S bacterial load did not change between the baseline and IVR removal visits for women in any arm (Fig. 1A; TFV/LNG: log_10_8.29 vs log_10_8.14, p = 0.56; TFV: log_10_7.84 vs log_10_7.94, p = 0.36; placebo: log_10_8.14 vs log_10_8.45, p = 0.25) and the difference in the degree of change across arms relative to the placebo arm was small (Fig. 1B). At IVR removal, no participant-level differences were observed in any arms between the lateral vaginal wall bacterial load and that of the IVR surface (Fig. S1; TFV/LNG: log_10_8.14 vs log_10_8.56, p = 0.11; TFV: log_10_7.94 vs log_10_8.22, p = 0.43; placebo: log_10_8.45 vs log_10_8.18, p = 0.25). Compared to the placebo arm, a higher estimated bacterial load was found on the TFV/LNG IVR surface (median difference of log_10_0.33, p = 0.05) than on the TFV IVR (median difference of log_10_0.04, p = 0.20 vs placebo median difference of log_10_− 0.34, Fig. 1C) relative to the vaginal wall bacterial load. Overall, genital bacterial load was positively correlated with the total abundance of the *Lactobacillus* genus, with a log_10_0.95 increase in total bacterial load with every log_10_ increase in *Lactobacillus* abundance (Linear regression p = 0.03 after adjusting for multiple comparisons).

### Change in bacterial load was not dependent on length of IVR use

**Figure 2:**
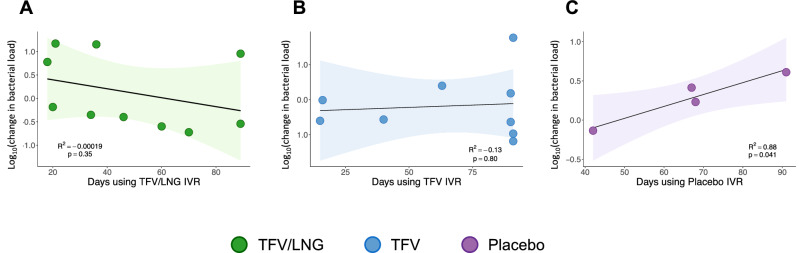
Impact of length of tenofovir/levonorgestrel (TFV/LNG), tenofovir (TFV), or placebo intravaginal ring (IVR) use on total bacterial load, Kisumu, Kenya, 2019. Linear regressions showing the association between number of days of IVR use and change in bacterial load between visits. The coloured shading represents the 95% confidence interval around the slope.

There was no association between the vaginal bacterial load and the number of days a woman used the TFV/LNG IVR (Slope =  − 0.009; 95% CI [− 0.032, 0.012]; p = 0.34; adjusted coefficient of determination (R^2^) =  − 0.00019) or the TFV IVR (Slope = 0.003; 95% CI [− 0.031, 0.012]; p = 0.80; adjusted R^2^ =  − 0.13). Women using the placebo IVR had a greater estimated increase in bacterial load with longer IVR use (Fig. 2; R^2^ = 0.88) although this estimate is highly imprecise since only 4 women were in this arm. Similarly, the correlation between IVR surface bacterial load and length of time of IVR use was low for either arm (Fig. S2).

### The overall microbiota diversity did not change with IVR use

**Figure 3:**
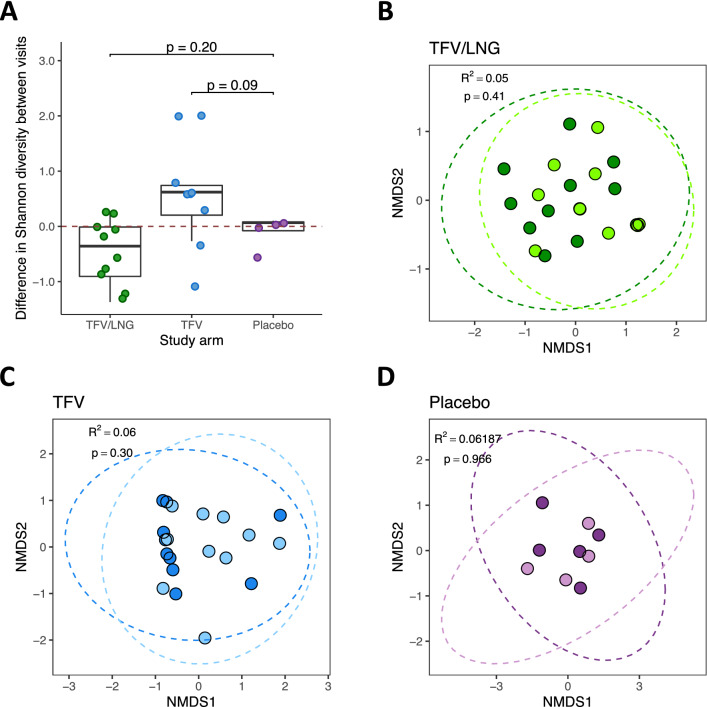
No change in overall microbiota diversity with use of tenofovir/levonorgestrel (TFV/LNG), tenofovir (TFV), or placebo intravaginal ring (IVR), Kisumu, Kenya, 2019. (**A**) Comparing the change in Shannon diversity (within-participant diversity) with each IVR. The dotted red line at zero indicates no change over time. (**B–D**) Principal component analysis plots showing the overlap between samples at baseline and the IVR removal visit for the TFV/LNG, TFV and Placebo IVRs, based on Bray–Curtis distances (between-participant diversity). p values were determined using the (**A**) Mann–Whitney U test or the (**B–D**) Adonis/PERMANOVA test based on permutations of distance matrices. *NMDS* non-metric multidimensional scaling.

To measure the effect of IVR use on the genital microbial composition of women, bacterial diversity was assessed using the Shannon diversity index and Bray–Curtis distances. We did not find a large change in overall microbiota diversity between baseline and IVR removal with use of any IVR (Fig. 3A–D). Microbial diversity decreased in women using the TFV/LNG IVR and increased with the TFV IVR (Shannon index difference of − 0.36 and 0.62 respectively; Fig. 3A); however both changes were not statistically significant. We also found no difference in the microbial diversity of the IVR microbiota compared to the vaginal microbiota at the IVR removal visit (Fig. S3; PERMANOVA TFV/LNG R^2^ = 0.058, p = 0.32; TFV R^2^ = 0.011, p > 0.99; placebo R^2^ = 0.11, p = 0.57).

### TFV/LNG IVR use was associated with a shift towards a less diverse CST

Based on hierarchical clustering of their dominant vaginal microbiota taxa, women were categorized as having community state type (CST) I if they had a genital microbiota dominated by *L. crispatus*, or CST III if their microbiota was dominated by *L. iners*. CST IVA had a higher abundance of the BV-associated bacterium *G. vaginalis* and CST IVB was composed of a broader range of BV-associated bacteria (Fig. S4). We found some discordance between Nugent-BV status and CST IV categorization. At baseline, of the 11/26 (42.3%) of women with a microbiota typically associated with microbial dysbiosis (CST IV A/B), 3/11 were Nugent-BV-positive, 3/11 had an intermediate Nugent score and 5/11 were Nugent-BV-negative. Further one participant with Nugent-BV was categorized as having a CST III microbiota.

**Figure 4:**
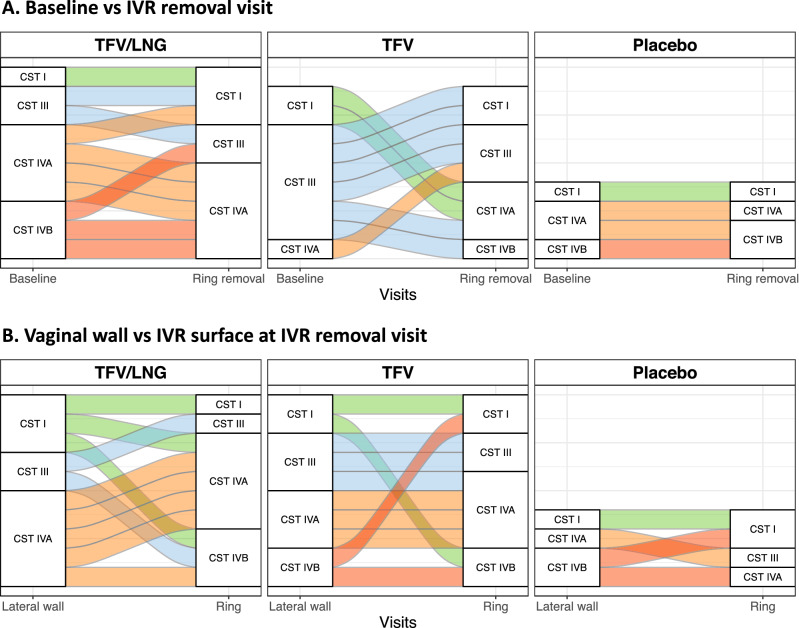
Transitions in bacterial communities within each study arm, Kisumu, Kenya, 2019. (**A**) Shifts in community state types (CSTs) in the genital tract from baseline to the intravaginal ring (IVR) removal visit. (**B**) Differences in CST between the genital tract and the IVR surface at the IVR removal visit. Each line represents one participant’s transition within the tenofovir/levonorgestrel (TFV/LNG), tenofovir (TFV), or placebo study arms.

During follow up, 50.0% (5/10) of women using the TFV/LNG IVR transitioned to a CST less associated with BV (2/5 transitioned from CST IVB to CST IVA) or a higher abundance of *Lactobacillus* species (1/5 transitioned from CST IVB to CST III, 1/5 from CST IVA to CST I, and 1/5 from CST III to CST I) while there were no CST shifts in the other 50.0% of women (Fig. 4A, Table S1). Among women using the TFV IVR, 22.2% (2/9) of women did not experience CST shifts, while the majority transitioned to a more diverse (44.4%; 4/9) or a less diverse CST (33.0%; 3/9). With the placebo IVR, 75.0% of women (3/4) did not transition to a different CST and one participant transitioned to a more diverse CST (CST IVA to CST IVB).

Overall, 41.6% (10/24) women had differences in assigned CST between the vaginal wall and the IVR surface at IVR removal (Fig. 4B), with 6/10 having a more diverse and 4/10 having a less diverse IVR CST, although none of these broad transitions were statistically significant (TFV/LNG p = 0.58; TFV p = 0.57; placebo p = 0.85).

### Neither TFV/LNG nor TFV IVR use was associated with major increases in pathogenic bacterial taxa

**Figure 5:**
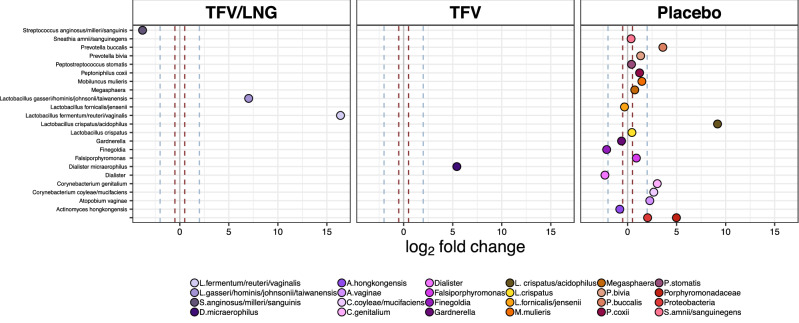
Fold changes in abundance of specific bacteria with the tenofovir/levonorgestrel (TFV/LNG), tenofovir (TFV), and placebo intravaginal rings (IVRs) respectively, Kisumu, Kenya, 2019. Dots on the right hand side of the gray solid line show a fold change increase in bacterial abundance and dots on the left hand side show a fold change decrease. The red and blue dotted lines represent 0.5-fold and twofold changes in bacterial abundance. Differential abundances with an adjusted p value of ≤ 0.01 are shown.

Longitudinal fold-changes in relative abundances of each individual bacterial taxa measured was determined for each study arm, accounting for individual variations in time between IVR insertion and removal. Using a threshold cutoff of log_2_ 0.05, the placebo IVR was associated with the most fluctuations, with a significant fold-change in 23 bacterial taxa (Fig. 5, Table S2): including decreases in *Finegoldia* spp. and *Dialister* spp. (~ log_2_ twofold), and increases in numerous taxa such as a > log_2_ twofold increase in *Proteobacteria phylum, Atopobium vaginae, Corynebacterium coyleae/mucifaciens, Corynebacterium genitalium, Prevotella buccalis*, and *Porphyromonadaceae* as well as a log_2_ 9.17-fold (standard error (SE) = 2.5) increase in *L. crispatus/acidophilus*. Women randomized to the TFV/LNG and TFV IVRs appeared to experience a more ‘stabilizing’ effect, with less change in vaginal bacterial taxa. Women using the TFV IVR were observed to have an average 5.42-fold (SE = 1.63) increase in *Dialister micraerophilus* abundance, a BV-associated bacterium. Women using the TFV/LNG IVR were observed to have a 3.79-fold (SE = 1.55) decrease in the pathogenic bacterium *Streptococcus anginosus/milleri/sanguinis* and a 16.3-fold (SE = 2.09) and 7.0-fold (SE = 2.13) increases in *L. fermentum/reuteri/vaginalis* and *L. gasseri* respectively (all p ≤ 0.01; Fig. 5).

### *Candidiasis* was similar across IVR types

**Figure 6:**
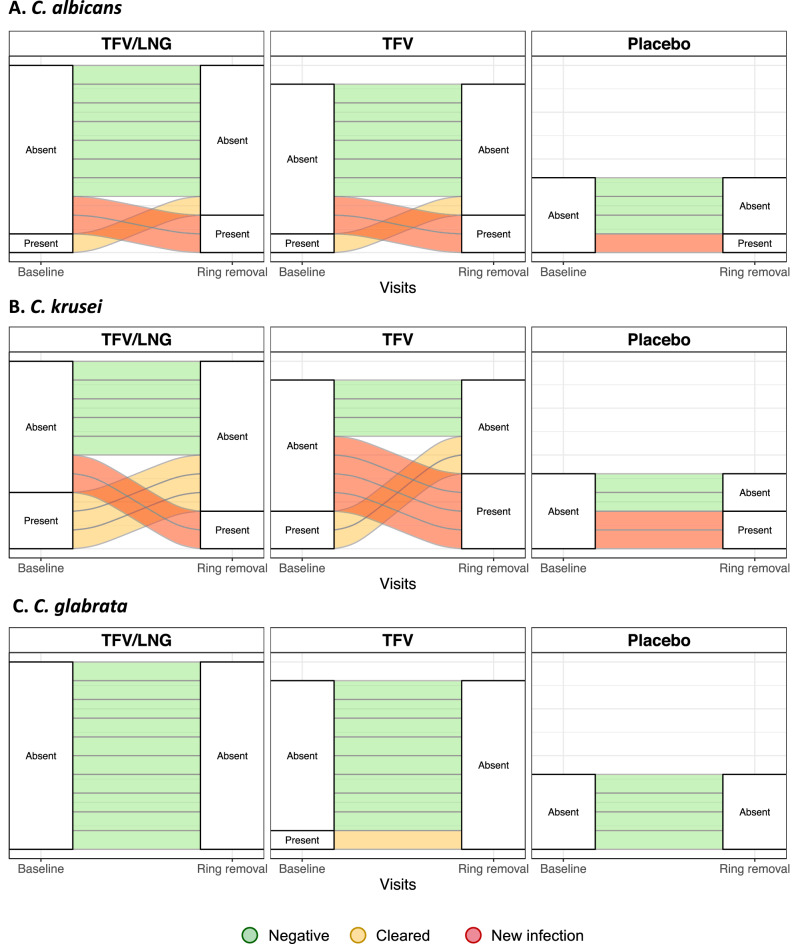
Longitudinal *Candida* status within each study arm for (**A**) *C. albicans,* (**B**) *C. krusei,* and (**C**) *C. glabrata*, Kisumu, Kenya, 2019. Women who remained *Candida-*negative are shown in green. Cleared infections are shown in yellow and new infections are shown in red.

The prevalence of *Candida* spp. were also measured at both visits. No *C. parapsilosis* was detected in any sample*.* There was an overall 30.8% prevalence of *Candida* at baseline and 44% at the IVR removal visit (p = 0.54; Table 2). *C. krusei* (23%) and *C. albicans* (17%) were more common (Table 2, Fig. 6). The distribution of each *Candida* spp. did not differ across study arms at baseline (*C. albicans* p > 0.99; *C. glabrata* p = 0.57; *C. krusei* p > 0.99) or at IVR removal (*C. albicans* p > 0.99; *C. krusei* p = 0.64). *C. glabrata* was not detected at the IVR removal visit. There were no differences in the overall microbial diversity (PERMANOVA p = 0.07) or differences in abundances of individual bacterial taxa between women who remained *Candida*-negative and those who acquired a *Candida* infection by the IVR removal visit.

**Table 2:** Prevalence of *Candida* species at baseline and removal of tenofovir/levonorgestrel (TFV/LNG), TFV or placebo intravaginal ring (IVR), Kisumu, Kenya, 2019.

	TFV/LNG		TFV		Placebo	
	Baseline n/N (%)	IVR removal n/N (%)	Baseline n/N (%)	IVR removal n/N (%)	Baseline n/N (%)	IVR removal n/N (%)
All *Candida* species	4/10 (40.0%)	4/10 (40.0%)	3/9 (33.3%)	4/9 (44.4%)	0/4 (0.0%)	2/4 (50.0%)
***C. albicans***						
Prevalence	1/10 (10.0%)	2/10 (20.0%)	1/9 (11.1%)	2/9 (22.2%)	0/4 (0.0%)	1/4 (25.0%)
Mean concentration (log_10_ copies/swab)	5.051	4.098	2.345	4.307	–	2.478
***C. krusei***						
Prevalence	3/10 (30.0%)	2/10 (20.0%)	2/9 (22.2%)	4/9 (44.4%)	0/4 (0.0%)	2/4 (50.0%)
Mean concentration (log_10_ copies/swab)	5.392	2.081	3.371	6.561	–	2.224
***C. glabrata***						
Prevalence	0/10 (0.0%)	0/10 (0.0%)	1/9 (11.1%)	0/9 (0.0%)	0/4 (0.0%)	0/4 (0.0%)
Mean concentration (log_10_ copies/swab)	–	–		–	–	–

Only samples for which matched longitudinal data was available were included.

### Higher microbial diversity was associated with lower TFV levels

**Figure 7:**
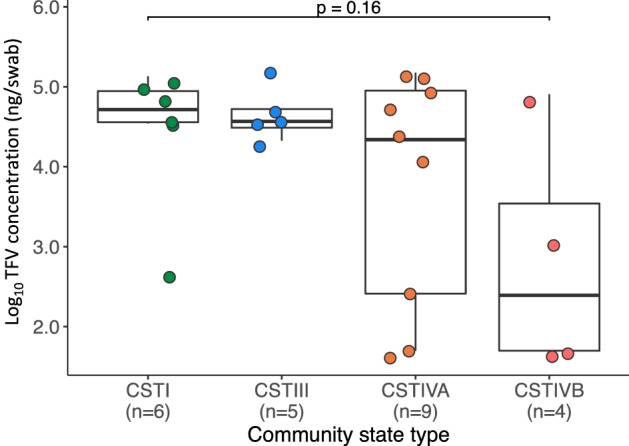
Vaginal tenofovir (TFV) concentrations across community state types (CSTs), Kisumu, Kenya, 2019. Comparing the difference in log_10_ vaginal TFV concentrations at the IVR removal visit among women using the TFV and tenofovir/levonorgestrel arms categorized by their CST. The p value was determined using the Mann–Whitney U test.

At the IVR removal visit, we found a higher estimated concentration (84.6-fold) of vaginal TFV in women with lower microbial diversity (median 53550 ng/swab with CST I vs 632.5 ng/swab with CST IVB; p = 0.16; Fig. 7), although sample sizes were small. In addition, after adjusting for the number of days of IVR use, a log_10_ increase in the absolute abundance of *Lactobacillus* genus was associated with a log_10_ 0.41 ng/swab increase in TFV concentrations (p > 0.99).

## Discussion

Overall, we found no evidence that either the TFV/LNG or TFV IVR significantly affected a woman’s genital bacterial or candidal composition. Our results are consistent with previous findings, including a recent safety study of the same IVR among women in the US and Dominican Republic^16^.

We mostly found small longitudinal changes in total bacterial load or in the overall microbial diversities in all study arms, suggesting that these IVRs do not induce major shifts in the genital microbiota. At the IVR removal visit, CSTs between the lateral vaginal wall and the IVR did not differ, similar to previous findings^16^. Despite no CST changes in most women using the placebo IVR, its use led to fluctuations in multiple bacterial taxa, with statistically significant fold changes in both *Lactobacillus* spp. and BV-associated bacteria. The TFV IVR was only associated with an increase in *D. micraerophilus* abundance, a BV-associated bacterium strongly associated with microbial diversity and high-inflammation states^17–19^. However, we did not see a shift to a more BV-associated microbial composition, likely because the increase in *D. micraerophilus* was insufficient to drive this change. Interestingly, there was a statistically significant decrease in *S. anginosus/milleri/sanguinis* abundance with the TFV/LNG IVR and a markedly larger fold-increase in *Lactobacillus* abundance, suggesting a positive effect of the TFV/LNG combination on microbial composition. Previous studies of vaginal rings found no significant destabilization of the genital microbiota, and an increase in *Lactobacillus* spp. in some cases^16,20,21^, which was attributed to ethinyl estradiol^22–24^. Here we found a similar stabilizing effect, with a decrease in microbial diversity with the TFV/LNG IVR despite containing LNG, a progestin, indicating that the decrease in microbial diversity and increase in *Lactobacillus* spp. could be associated with progestin use, particularly LNG, in line with a previous study reporting an increase in genital *Lactobacillus* spp. concentration with LNG implant use^25^.

Although not statistically significantly so, we found that genital TFV concentrations at the IVR removal visit were positively associated with *Lactobacillus* spp. abundances. This is similar to other studies showing that TFV-based HIV PrEP products were less effective in *Lactobacillus*-deficient states^14^. There was a decreasing trend in microbial diversity with increased TFV concentration, with a 84.6-fold higher TFV concentration found with an *L. crispatus*-dominant microbiota (CST I) compared to a more diverse BV-associated microbiota (CST IVB). Based on our differential abundance analyses, this was not driven *by G. vaginalis* or *Prevotella* spp as previously described^14,26^. More work is needed to determine if this decrease is clinically relevant to impact the IVR capacity to prevent HIV or HSV-2. In particular, it would be important to assess the relationship between CSTs and concentrations of the TFV active metabolite, TFV-diphosphate, in mucosal tissues.

A primary limitation was the small sample size, due to the study being a phase-IIa safety trial. Thus, we presented substantial descriptive data that examine actual numbers and trends, rather than just statistical comparisons. More in-depth comparisons, in larger cohorts, that also include women with BV at IVR insertion, might better help identify potential genital microbial changes induced by these IVRs.

In this study measuring the safety of TFV/LNG and TFV polyurethane IVRs on the genital microbiota among women living in Kenya, we found no evidence of adverse changes to genital microbial health with up to 90 days of use. We found a trend towards the establishment of high *Lactobacillus* states with the TFV/LNG IVR, which could positively impact genital health in a population with a high BV prevalence^27–29^. These results indicate that TFV/LNG and TFV IVRs are likely safe to use and support further studies assessing their efficacy in preventing HIV, HSV-2, and unintended pregnancy.

## Methods

### Participant recruitment

CONRAD B17-144 was a phase-IIa randomised, placebo-controlled, investigational new-drug-enabling trial assessing the safety, pharmacokinetics, pharmacodynamics, tolerability, acceptability of, and adherence to two 90 days IVRs. Women were randomised in a 2:2:1 ratio to receive a TFV-only IVR^30^ to prevent HIV/HSV, a combination TFV/LNG IVR that additionally may prevent pregnancy^30–32^ and a placebo IVR^31^. All rings were composed of polyurethane tubing^31^.

This study recruited women at the Jaramogi Oginga Odinga Referral Hospital, Kisumu, Kenya, who were generally healthy, non-pregnant, at lower risk for HIV (based on a validated risk score^33^), seronegative for HIV and hepatitis B surface antigen, and bacterial vaginosis (BV)-negative by Amsel criteria. Women diagnosed with BV by Amsel criteria during the screening procedures were treated and permitted to be re-assessed for eligibility > 2 months after treatment, and enrolled if BV was not detected at that time. Eligible women were not using hormonal contraception at enrolment and had not been diagnosed or treated for STIs in the last three months (Supplementary Methods). The study was approved by the Scientific and Ethics Review Unit at the Kenya Medical Research Institute and the Human Subjects Division at University of Washington, and was registered with ClinicalTrials.gov (Identifier: NCT03762382; registered 03/12/2018). All participants provided written informed consent and all methods were carried out in accordance with relevant guidelines and regulations.

### Study procedures

Results from the parent study will be published in a separate manuscript (Mugo et al*.*^32^, in preparation). Paired data from the baseline/IVR insertion and IVR removal visits were included for this analysis, focused on microbiota changes. IVR removal was scheduled for 90 days after IVR insertion or prior to the IVR expiry date, whichever date came first (At 90 days: 12/27; before expiry date: 9/27; other reasons including pregnancy or STIs: 6/27). In cases where matched clinical data from the IVR removal visit were not available, samples collected during the next visit, which was scheduled to occur 24 h later, were used for analyses.

### Sample collection at IVR insertion and removal

After randomization, the IVR was inserted deeply into the vagina by a study clinician at the baseline visit and removed at the IVR removal visit. At both baseline and the IVR removal visits, vaginal swabs were collected for (1) TFV PK measurements, (2) Nugent score and vaginal pH and (3) microbiota 16S rRNA sequencing. A swab of the IVR surface was also collected at the IVR removal visit. Nugent-BV was defined as having a Nugent score ≥ 7; women with a Nugent score 4–6 were categorized as having an intermediate vaginal microbiota (Supplementary Methods).

### Genital TFV concentrations

TFV concentrations from cervicovaginal fluid from swabs were determined using liquid chromatography tandem mass spectrometry as described previously^34^ (Supplementary Methods).

### Microbial DNA extraction

DNA extraction from lateral vaginal wall swabs was carried out (Qiagen AllPrep PowerViral^®^ DNA/RNA kit; Supplementary Methods) and was stored at − 20 °C until used for 16S rRNA sequencing, total 16S bacterial load using real-time polymerase chain reaction (qPCR), and *Candida* spp. quantitation (qPCR).

### 16S rRNA gene sequencing and analysis

The V3–V4 hypervariable region of the bacterial 16S rRNA was amplified using modified universal primers^35^. Samples were sequenced using the Illumina MiSeq platform and (300 bp paired-end). DADA2 v1.12.1^36^ was used to process, merge and filter raw reads and samples with < 2000 reads were excluded from further analyses. Taxonomic annotation was carried out using the RDP database (v11.5) and a BLAST search was carried out for further taxonomic classification of unannotated amplicon sequence variants (ASV). Taxonomic annotation was available for 993 ASVs, with 277 (27.9%) having species-level annotation. The ASVs were merged at the lowest available taxonomic level to generate relative abundances for a total of 105 bacterial taxa^17^ (Supplementary Methods).

The total bacterial load per swab was determined using a qPCR of the 16S rRNA gene^37^. The BactQuant assay targets the V3–V4 region, and gives an estimate of the total 16S rRNA copies per swab. Based on these values and the relative abundances of taxa generated by 16S rRNA sequencing, absolute bacterial abundances (copies/swab) were calculated for each taxon. One sample (out of five), in the placebo arm, without sufficient 16S DNA to pass quality control checks was excluded from analysis (final n = 4). All further analyses were carried out using absolute abundances for bacterial taxa.

### *Candida* spp. quantification

Concentrations of *C. albicans, C. glabrata, C. krusei* and *C. parapsilosis,* were measured using species-specific qPCRs at the baseline and IVR removal visits (Supplementary Methods). The final concentrations were reported as number of log_10_ copies per swab.

### Statistical analysis

All microbiota data analysis was carried out in R (v3.6.0). Paired comparisons between the ring insertion and ring removal visits were considered primary endpoints of this analysis. Participant microbiota were categorised into four CSTs using Ward clustering of weighted Unifrac dissimilarity matrices derived from bacterial absolute abundances. TFV and TFV/LNG study arms were compared to the placebo arm using Mann–Whitney U-tests for independent samples and permutational multivariate analysis of variance (PERMANOVA) for microbial diversity. Paired analyses comparing microbiota at IVR insertion and removal visits were carried out using Wilcoxon Signed Rank tests and generalized estimating equation models. Correlations between bacterial absolute abundance and variables such as time of IVR use and genital TFV concentrations were estimated using linear regression models. The number of days of IVR use was categorized as ≤ 47 days (< 2 menstrual cycles), 48–71 days (~ 2 menstrual cycles) and 72–95 days (~ 3 menstrual cycles), and was adjusted for when measuring fold changes in individual bacterial taxa within study arms, using generalized linear models as part of the DeSeq2 package. Log_2_ fold-changes were determined only for bacterial taxa found in at least 15% of samples to minimize the influence of minority taxa. Due to the small sample size, the study was not powered to estimate differences between the study arms with a high degree of precision and analyses were descriptive in nature. As a result, p-values and confidence intervals (CI) were not adjusted for multiple comparisons unless explicitly stated, in which case a false discovery rate step down procedure was used^38^ (Supplementary Methods).

## Supplementary Information

Supplementary Information 1.

Supplementary Information 2.

Supplementary Figure S1.

Supplementary Figure S2.

Supplementary Figure S3.

Supplementary Figure S4.

## Data Availability

The dataset generated and analyzed in this study has been deposited at NCBI SRA under Accession ID PRJNA834833 (https://www.ncbi.nlm.nih.gov/bioproject/PRJNA834833). Linked data are available from the corresponding author on reasonable request.
