# Supplementary material for: Genital microbiota of women using a 90 day tenofovir or tenofovir and levonorgestrel intravaginal ring in a placebo controlled randomized safety trial in Kenya
Source: Sci Rep. 2022 Jul 14;12:12040. doi: 10.1038/s41598-022-13475-9 (PMC9283538; doi:10.1038/s41598-022-13475-9)
Supplement: Supplementary file 1 — Supplementary Information 1. [file 41598_2022_13475_MOESM1_ESM.docx]

**Genital microbiota of women using a 90 day tenofovir or tenofovir and levonorgestrel intravaginal ring in a placebo controlled randomized safety trial in Kenya**

Smritee Dabee^1^, Nelly Mugo^2,3^, Victor Mudhune^4^, Eleanor McLellan-Lemal^5^, Sue Peacock^2^, Siobhan O’Connor^5^, Betty Njoroge^3^, Beatrice Nyagol^4^, Andrea R. Thurman^6^, Eunice Ouma^4^, Renee Ridzon^5,7^, Jeffrey Wiener^5^, Harald S. Haugen^2^, Melanie Gasper^1^, Colin Feng^1^, Shannon A. Allen^8^, Gustavo F. Doncel^6^, Heather B. Jaspan^1,2,9^, Renee Heffron^2^, and Kisumu Combined Ring Study Team*

^1^Seattle Children’s Research Institute, Seattle, WA, USA; ^2^University of Washington Global Health, Seattle, WA USA; ^3^Kenya Medical Research Institute, Center for Clinical Research, Nairobi, Kenya; ^4^Kenya Medical Research Institute, Center for Global Health Research, Kisumu, Kenya; ^5^Centers for Disease Control and Prevention, Division of HIV/AIDS Prevention, Atlanta, GA, USA; ^6^CONRAD, Eastern Virginia Medical School, Norfolk, VA, USA; ^7^US National Institutes of Health, Bethesda, MD, USA; ^8^United States Agency for International Development, Washington, DC, USA; ^9^Institute of Infectious Diseases and Molecular Medicine, University of Cape Town, Cape Town, South Africa

## Participant recruitment and eligibility criteria

As part of CONRAD B17-144, a phase-IIa randomised placebo-controlled trial (Dec 2018 – Aug 2019), women were randomised to receive a polyurethane intravaginal ring (IVR) containing either (1) tenofovir (TFV) and levonorgestrel (LNG) (1.15g TFV/6.0mg LNG releasing ~10mg TFV and 20μg LNG daily), (2) TFV only (1.41g TFV releasing ~10mg TFV daily) or (3) placebo (non-eluting modified starch).

Women recruited for the study were generally healthy, non-pregnant, at lower risk for HIV infection, seronegative for hepatitis B surface antigen and negative for bacterial vaginosis (BV) by Amsel criteria. Eligible women did not have any contraindications to the active study agents, including TFV, LNG or excipient ingredients, were not using hormonal contraception at the time of trial enrolment, were willing to use use non-spermicidal condoms during the study, had not been diagnosed or treated in the last three months for any STI or pelvic inflammatory disease, and would not be using any product containing tenofovir, other than the study product. IVR use was discontinued early if women became pregnant, had a positive HIV/STI test, or were reported to have menorrhagia or vulvovaginitis. There were more removals due to menstrual bleeding changes with TFV/LNG IVR compared to the placebo or TFV-only IVRs.

## Study design and samples collected

Women attended a total of 10 visits following enrolment (Mugo *et al.* 2021, in preparation) but only data from the baseline/IVR insertion and IVR removal visits were included in this analysis. At the enrollment visit, women were randomized using a process with sequentially allocated randomization envelopes. The next available envelope (which had been prepared and sealed by the data center in the US) was opened and revealed an IVR code. The pharmacist then selected the next available IVR corresponding to the assigned code and provided that to the clinician (clinician-blinded) to carry out the insertion.

At baseline/IVR insertion, the IVR was placed at least 2 cm above the introitus, beyond the levator ani muscle. Instructions were given to the participant about how to replace the IVR themselves or come into the study clinic for replacement in case of accidental slippage or expulsion before the scheduled IVR removal visit.

After removal, the IVR was stabilized using sterile forceps and the surface was swabbed, avoiding the joint of the IVR, to assess microbial growth (ESwab^TM^ Copan Diagnostics). Dates and times of all IVR removals, discontinuations and expulsions were captured on electronic case report forms using self-reported information with clinical confirmation when possible.

At both visits, BV was determined using Amsel’s criteria as well as Nugent scoring by wet mount microscopy, and the vaginal pH was measured, from the lower lateral vaginal wall fluid (Dacron swab). The absence of Nugent-BV was defined as having a Nugent score ≤3, while women with a Nugent score 4-6 were classified as having an intermediate vaginal microbiota and those with a Nugent score ≥7 as having Nugent-BV.

### TFV concentrations

Liquid chromatography tandem mass spectrometry was used to measure genital TFV concentrations from Dacron swab cervicovaginal fluid. Briefly, an isotopically-labeled internal standard was added to the swabs as previously described^1^, and centrifuged into a collection tube. Samples were processed using an optimised protocol as described previously^2^ [Mugo *et al.* 2021, in preparation]. The analytical measuring ranges of TFV in cervicovaginal fluid collected on Dacron swabs were 0.5-2000 ng/mL or 0.05-200 ng/sample.

### Microbiome swab collection and microbial DNA extraction

Vaginal swabs (lower lateral vaginal wall) and IVR swabs (IVR surface; both ESwab^TM^ Copan Diagnostics) were slowly thawed in their collection tubes, containing transport media, on ice at 4^o^C. The samples were gently shaken at 100rpm for 3 minutes to obtain a homogeneous bacterial suspension, of which 200μl was used for microbial DNA extraction (Qiagen AllPrep PowerViral^®^ DNA/RNA kit) according to manufacturer’s instructions. DNA was stored at -20^o^C until used for 16S rRNA sequencing as well as total 16S bacterial load, and *Candida* spp. quantitation using real-time polymerase chain reaction (qPCR).

### 16S rRNA sequencing and total bacterial load

All samples were run in duplicates. Modified universal primers described by Gohl et al. (2016) were used to amplify the V3-V4 hypervariable region of the bacterial 16S rRNA^3^, except using 357F/806R primers (Table S3). The amplified products were pooled and cleaned using Agencourt AMPure XP beads (Beckman Coulter; CA, USA). Dual-index barcodes and Illumina sequencing adapters were attached using the Nextera XT DNA Prep kit (Illumina; CA, USA). Amplicons were pooled in equimolar amounts to be sequenced using V3 600 cycle kits on the Illumina MiSeq platform and (300bp paired-end). Following demultiplexing, raw reads were preprocessed, merged, and filtered using DADA2 v1.12.1^4^. Using a custom python script, primer sequences were removed and the reads truncated at 250bp. A sample threshold cutoff of 2000 reads was applied.

### Measurement of *Candida* spp. concentrations

DNA extracted from lateral wall swabs was used to measure the concentrations of four candida species; *Candida albicans, C. glabrata, C. krusei* and *C. parapsilosis* at both the baseline and IVR removal visits*.* Applied Biosystems TaqMan™ Vaginal Microbiota qPCR assays (ThermoFisher) were used for each of the four species. Absolute quantities were extrapolated from a standard curve generated using a multi-target plasmid (TaqMan™ Vaginal Microbiota Amplification Control), which includes amplicons for each *Candida* spp. The final concentrations were reported as number of log_10_ copies per swab.

### Statistical analyses

All downstream analyses were carried out in R (v3.6.0). The packages phyloseq ^5^, NMF ^6^, metagenomeSeq^7^, vegan ^8^, and DESeq2^9^ were used for microbiota data analysis including ecological diversity analyses, ordination and differential abundance calculations of specific bacterial taxa from IVR insertion to removal.

Participant microbiota were categorised into four community state types (CSTs) as previously defined^10^, using the derived bacterial absolute abundances and Ward clustering of weighted Unifrac dissimilarity matrices. These clusters were categorized as CST I (Lactobacillus crispatus-dominant), CST III (L. iners-dominant), IVA (higher Gardernella vaginalis abundance) and IVB (highly diverse, BV-associated) based on their dominant vaginal microbiota taxa.

Mann-Whitney U-tests for independent samples were used to compare bacterial loads and TFV concentrations in both active arms (TFV-only and TFV/LNG IVRs) to the placebo arm. Wilcoxon Signed Rank tests for matched samples were used for comparison of baseline and IVR removal. A generalized estimating equation model was used to measure longitudinal associations between baseline and IVR removal. Using the adonis function within the vegan package, permutational multivariate analysis of variance (PERMANOVA) was used to compare microbial diversity in each active arm relative to the placebo arm. Bray-Curtis distances were used for this analysis and 999 permutations were specified. Linear regression models were used to determine the association between bacterial absolute abundance and variables such as time of IVR use and genital TFV concentrations.

## References

1. Haaland, R. E. *et al.* Antiretroviral drug exposure in urethral and glans surface sampling of the penis. *J. Antimicrob. Chemother.* **76**, 2368–2374 (2021).

2. Hendrix, C. W. *et al.* MTN-001: Randomized pharmacokinetic cross-over study comparing tenofovir vaginal gel and oral tablets in vaginal tissue and other compartments. *PLoS One* **8**, e55013 (2013).

3. Gohl, D. M. *et al.* Systematic improvement of amplicon marker gene methods for increased accuracy in microbiome studies. *Nat. Biotechnol.* **34**, 942–949 (2016).

4. Callahan, B. J. *et al.* DADA2: High-resolution sample inference from Illumina amplicon data. *Nat. Methods* **13**, 581–583 (2016).

5. McMurdie, P. J. & Holmes, S. phyloseq: an R package for reproducible interactive analysis and graphics of microbiome census data. *PLoS One* **8**, e61217 (2013).

6. Gaujoux, R. *Generating heatmaps for Nonnegative Matrix Factorization*. *R Foundation for Statistical Computing, Vienna, Austria* (2014).

7. Paulson, J. N., Stine, O. C., Bravo, H. C. & Pop, M. Differential abundance analysis for microbial marker-gene surveys. *Nat. Methods* **10**, 1200–2 (2013).

8. Oksanen, J. *et al.* vegan: Community Ecology Package. (2019).

9. Love, M. I., Huber, W. & Anders, S. Moderated estimation of fold change and dispersion for RNA-seq data with DESeq2. *Genome Biol.* **15**, 550 (2014).

10. France, M. T. *et al.* VALENCIA: a nearest centroid classification method for vaginal microbial communities based on composition. *Microbiome* **8**, 166 (2020).
