# Supplementary material for: Genital microbiota of women using a 90 day tenofovir or tenofovir and levonorgestrel intravaginal ring in a placebo controlled randomized safety trial in Kenya
Source: Sci Rep. 2022 Jul 14;12:12040. doi: 10.1038/s41598-022-13475-9 (PMC9283538; doi:10.1038/s41598-022-13475-9)
Supplement: Supplementary file 2 — Supplementary Information 2. [file 41598_2022_13475_MOESM2_ESM.docx]

**Genital microbiota of women using a 90 day tenofovir or tenofovir and levonorgestrel intravaginal ring in a placebo controlled randomized safety trial in Kenya**

Smritee Dabee^1^, Nelly Mugo^2,3^, Victor Mudhune^4^, Eleanor McLellan-Lemal^5^, Sue Peacock^2^, Siobhan O’Connor^5^, Betty Njoroge^3^, Beatrice Nyagol^4^, Andrea R. Thurman^6^, Eunice Ouma^4^, Renee Ridzon^5,7^, Jeffrey Wiener^5^, Harald S. Haugen^2^, Melanie Gasper^1^, Colin Feng^1^, Shannon A. Allen^8^, Gustavo F. Doncel^6^, Heather B. Jaspan^1,2,9^, Renee Heffron^2^, and Kisumu Combined Ring Study Team*

^1^Seattle Children’s Research Institute, Seattle, WA, USA; ^2^University of Washington Global Health, Seattle, WA USA; ^3^Kenya Medical Research Institute, Center for Clinical Research, Nairobi, Kenya; ^4^Kenya Medical Research Institute, Center for Global Health Research, Kisumu, Kenya; ^5^Centers for Disease Control and Prevention, Division of HIV/AIDS Prevention, Atlanta, GA, USA; ^6^CONRAD, Eastern Virginia Medical School, Norfolk, VA, USA; ^7^US National Institutes of Health, Bethesda, MD, USA; ^8^United States Agency for International Development, Washington, DC, USA; ^9^Institute of Infectious Diseases and Molecular Medicine, University of Cape Town, Cape Town, South Africa

## Supplementary tables

**Table S1.** **Change in CST distribution from baseline (rows) to ring removal (columns)*.**

|  |  | **Ring removal visit** | | | |
| --- | --- | --- | --- | --- | --- |
|  | **Placebo IVR** | CST I | CST III | CST IVA | CST IVB |
| **Baseline** | CST I | 1 | - | - | - |
|  | CST III | - | - | - | - |
|  | CST IVA | - | - | 1 | 1 |
|  | CST IVB | - | - | - | 1 |
|  | **TFV IVR** | CST I | CST III | CST IVA | CST IVB |
| **Baseline** | CST I | - | - | 2 | - |
|  | CST III | 2 | 2 | 1 | 1 |
|  | CST IVA | - | 1 | - | - |
|  | CST IVB | - | - | - | - |
|  | **TFV/LNG IVR** | CST I | CST III | CST IVA | CST IVB |
| **Baseline** | CST I | 1 | - | - | - |
|  | CST III | 1 | 1 | - | - |
|  | CST IVA | 1 | - | 3 | - |
|  | CST IVB | - | 1 | 2 | - |

The grey squares indicate no transition in CST, the yellow squares represent a transition to a more diverse CST, and the green cells are instances where the vaginal microbiota shifted to a less diverse CST. The numbers indicate the number of women in each cell.

**Table S2. Fold change differences in bacterial abundance at ring removal compared to baseline at the ring insertion visit, Kisumu, Kenya, 2019.**

| **Bacterial taxa** | **Study Arm** | **log_2_FoldChange** | **Standard error** | **p value^a^** | **Difference (log_2_ absolute abundance)^b^** |
| --- | --- | --- | --- | --- | --- |
| *Lactobacillus gasseri/hominis/johnsonii/taiwanensis* | TFV/LNG | 7.026 | 2.127 | 0.006 | 0.021 |
| *Streptococcus anginosus/milleri/sanguinis* | TFV/LNG | -3.793 | 1.556 | 0.006 | -0.868 |
| *Lactobacillus fermentum/reuteri/vaginalis* | TFV/LNG | 16.390 | 2.086 | 0.010 | 4.397 |
| *Dialister micraerophilus* | TFV | 5.422 | 1.627 | 0.014 | 5.966 |
| *Dialister* spp. | Placebo | -2.306 | 1.820 | 0.009 | -3.890 |
| *Finegoldia* spp*.* | Placebo | -2.130 | 1.989 | 0.003 | -0.626 |
| *Actinomyces hongkongensis* | Placebo | -0.793 | 1.909 | 0.001 | 0.27 |
| *Gardnerella* spp. | Placebo | -0.615 | 1.892 | 0.006 | 0.512 |
| *Lactobacillus fornicalis/jensenii* | Placebo | -0.311 | 2.132 | <0.001 | 0.234 |
| *Saccharofermentans BVAB3* | Placebo | -0.255 | 2.070 | <0.001 | 0.522 |
| *Peptoniphilus lacrimalis* | Placebo | 0.197 | 1.836 | 0.006 | 1.238 |
| *Fusobacterium nucleatum/periodonticum* | Placebo | 0.202 | 1.917 | 0.001 | 0.755 |
| *Sneathia amnii/sanguinegens* | Placebo | 0.360 | 1.830 | 0.004 | 0.636 |
| *Peptostreptococcus stomatis* | Placebo | 0.402 | 2.014 | <0.001 | -0.209 |
| *Lactobacillus crispatus* | Placebo | 0.445 | 2.535 | <0.001 | -0.485 |
| *Megasphaera* spp*.* | Placebo | 0.734 | 1.667 | 0.001 | 0.983 |
| *Falsiporphyromonas* | Placebo | 0.898 | 1.827 | 0.002 | 1.085 |
| *Peptoniphilus coxii* | Placebo | 1.226 | 1.745 | <0.001 | 0.283 |
| *Prevotella bivia* | Placebo | 1.334 | 2.497 | 0.002 | 0.193 |
| *Mobiluncus mulieris* | Placebo | 1.460 | 1.935 | 0.001 | 1.353 |
| *Proteobacteria phylum* | Placebo | 2.041 | 1.952 | 0.001 | 0.945 |
| *Atopobium vaginae* | Placebo | 2.266 | 2.054 | <0.001 | 0.555 |
| *Corynebacterium coyleae/mucifaciens* | Placebo | 2.673 | 2.578 | 0.004 | 0.852 |
| *Corynebacterium genitalium* | Placebo | 3.027 | 2.615 | 0.002 | 1.028 |
| *Prevotella buccalis* | Placebo | 3.599 | 2.047 | 0.002 | 3.144 |
| *Porphyromonadaceae family* | Placebo | 4.982 | 2.132 | 0.007 | 4.623 |
| *Lactobacillus crispatus/acidophilus* | Placebo | 9.173 | 2.455 | 0.002 | 7.02 |

^a^ p values based on a Wald test using parameters estimated by maximum likelihood, and adjusted for time of IVR use and multiple comparisons using the FDR/Benjamini-Hochberg method

^b^ Unadjusted difference in the mean log_2_ absolute abundance values between the ring insertion and ring removal visits (reported as log_2_ copies/swab)

**Table S3. Primers targeting the 16S rRNA gene V3-4 region**

| **Primer name** | **Sequence** |
| --- | --- |
| V4_806R_Nextera | GTCTCGTGGGCTCGGAGATGTGTATAAGAGACAGGG**ACTACNVGGGTWTCTAAT** |
| V3_357F_Nextera | TCGTCGGCAGCGTCAGATGTGTATAAGAGACAGCC**TACGGGNGGCWGCAG** |

## Supplementary figure legends

**FigS1. Bacterial load did not differ between the vaginal wall and intravaginal ring (IVR) surface at the IVR removal visit, Kisumu, Kenya, 2019.** Comparing the log_10_ total 16S copies between the lateral vaginal wall and IVR surface swabs for women using the tenofovir/levonorgestrel (TFV/LNG), tenofovir (TFV), and placebo IVRs. p values were determined using the Wilcoxon signed rank test.

**FigS2. Intravaginal ring (IVR) surface bacterial load did not depend on time of IVR use, Kisumu, Kenya, 2019.** Linear regression showing the association between number of days of IVR use and the bacterial load on the IVR surface. The coloured shading represents the 95% confidence interval around the slope.

**FigS3.** **No change in overall microbiota diversity between the lateral vaginal wall and intravaginal ring (IVR) surface at IVR removal, Kisumu, Kenya, 2019.** Principal component analysis plots showing the overlap between samples collected from the vaginal wall and IVR surface at the IVR removal visit, based on Bray-Curtis distances. p values were determined using the Adonis/PERMANOVA test based on permutations of distance matrices. NMDS: Non-metric Multidimensional Scaling

**FigS4.** **Distribution of bacterial taxa among the four community state types (CSTs), Kisumu, Kenya, 2019.** (A) 15 most abundant taxa in CST I *(Lactobacillus crispatus-*dominant), CST III *(L. iners-*dominant), CST IVA (Diverse, with higher abundance of *Gardnerella vaginalis*) and CST IVB (Highest diversity) clusters*.* (B) Principal component analysis plots showing the different CST clusters. (C) Distribution of the 15 most abundant taxa in all the sequenced samples. The p value was determined using the Adonis/PERMANOVA test based on permutations of distance matrices.
