# Supplementary figures and images for: Genital microbiota of women using a 90 day tenofovir or tenofovir and levonorgestrel intravaginal ring in a placebo controlled randomized safety trial in Kenya
Source: Sci Rep. 2022 Jul 14;12:12040. doi: 10.1038/s41598-022-13475-9 (PMC9283538; doi:10.1038/s41598-022-13475-9)

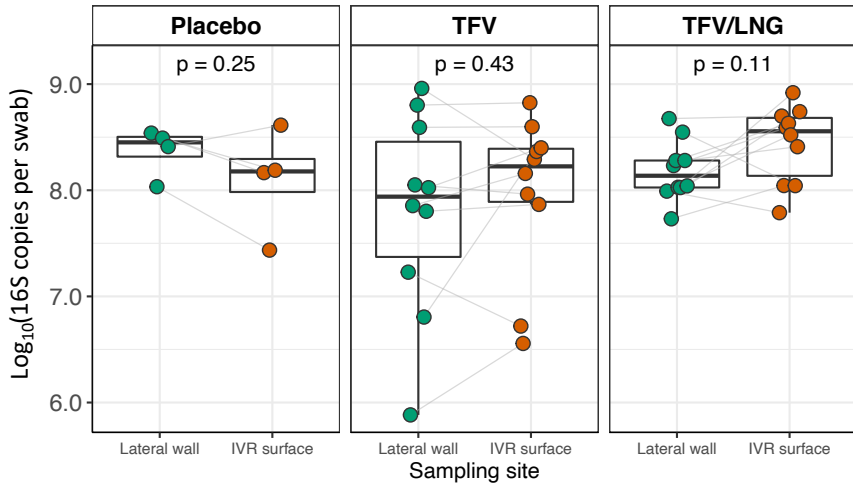

| IVR            | Lateral wall<br>Median [IQR] | IVR surface<br>Median [IQR] | p value |
|----------------|------------------------------|-----------------------------|---------|
| <b>TFV/LNG</b> | 8.14 [8.03-8.28]             | 8.56 [8.04-8.70]            | 0.11    |
| <b>TFV</b>     | 7.94 [7.23-8.59]             | 8.22 [7.87-8.40]            | 0.43    |
| <b>Placebo</b> | 8.45 [8.22-8.51]             | 8.18 [7.80-8.40]            | 0.25    |

Supplement: Supplementary file 3 — Supplementary Figure S1. [file 41598_2022_13475_MOESM3_ESM.pdf]

**A**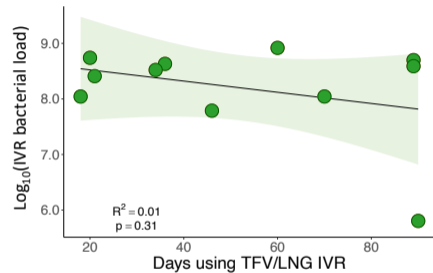**B**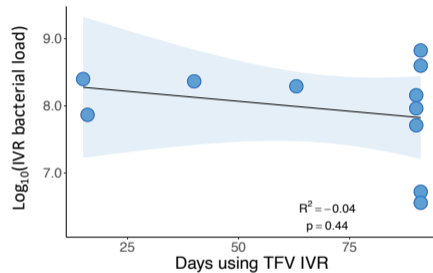**C**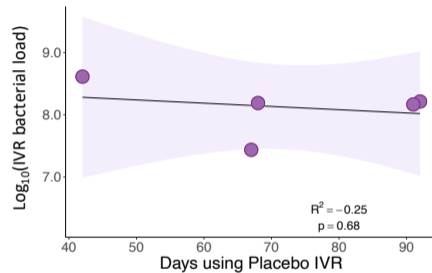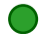

TFV/LNG

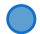

TFV

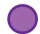

Placebo

Supplement: Supplementary file 4 — Supplementary Figure S2. [file 41598_2022_13475_MOESM4_ESM.pdf]

**A**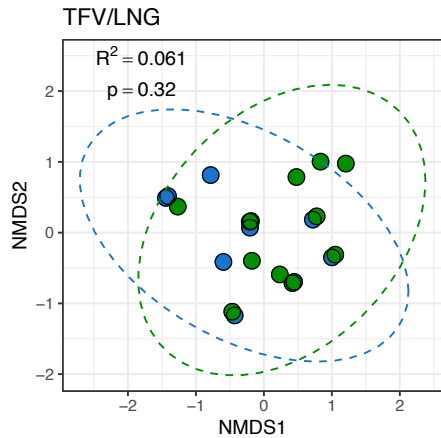**B**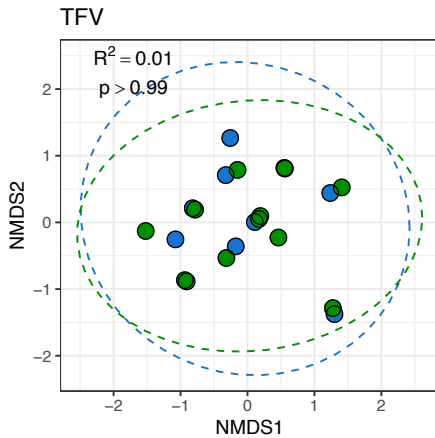**C**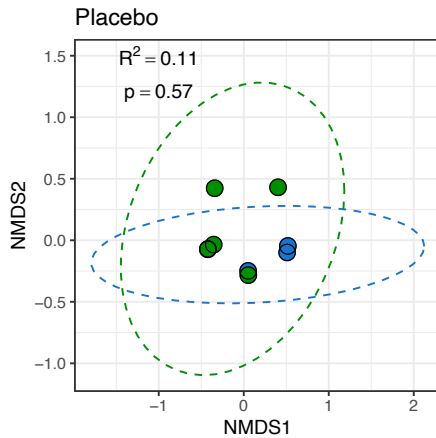

● Lateral wall    ● IVR surface

Supplement: Supplementary file 5 — Supplementary Figure S3. [file 41598_2022_13475_MOESM5_ESM.pdf]

**A**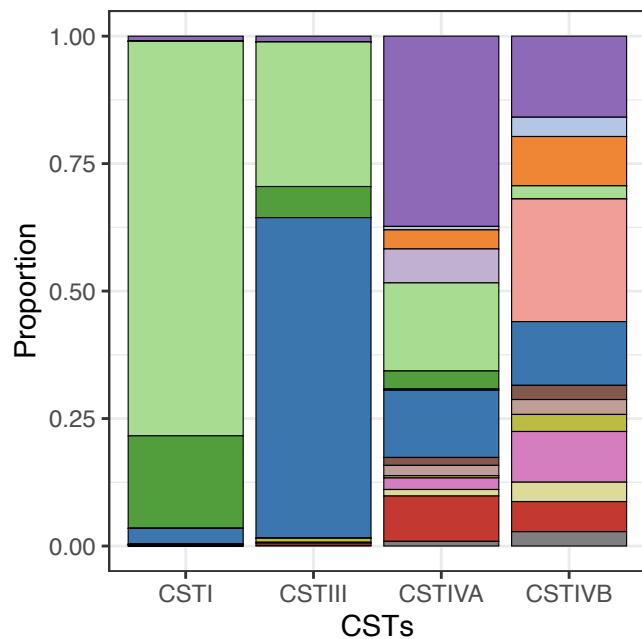**B**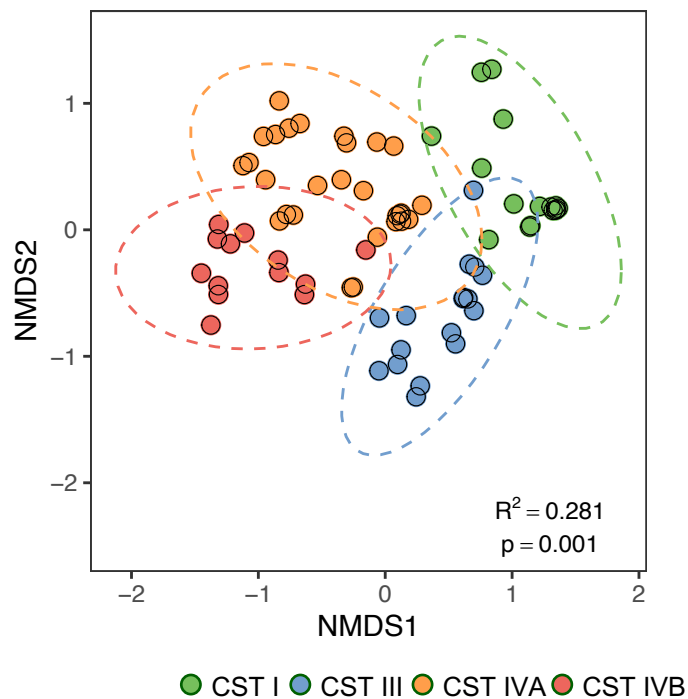**C**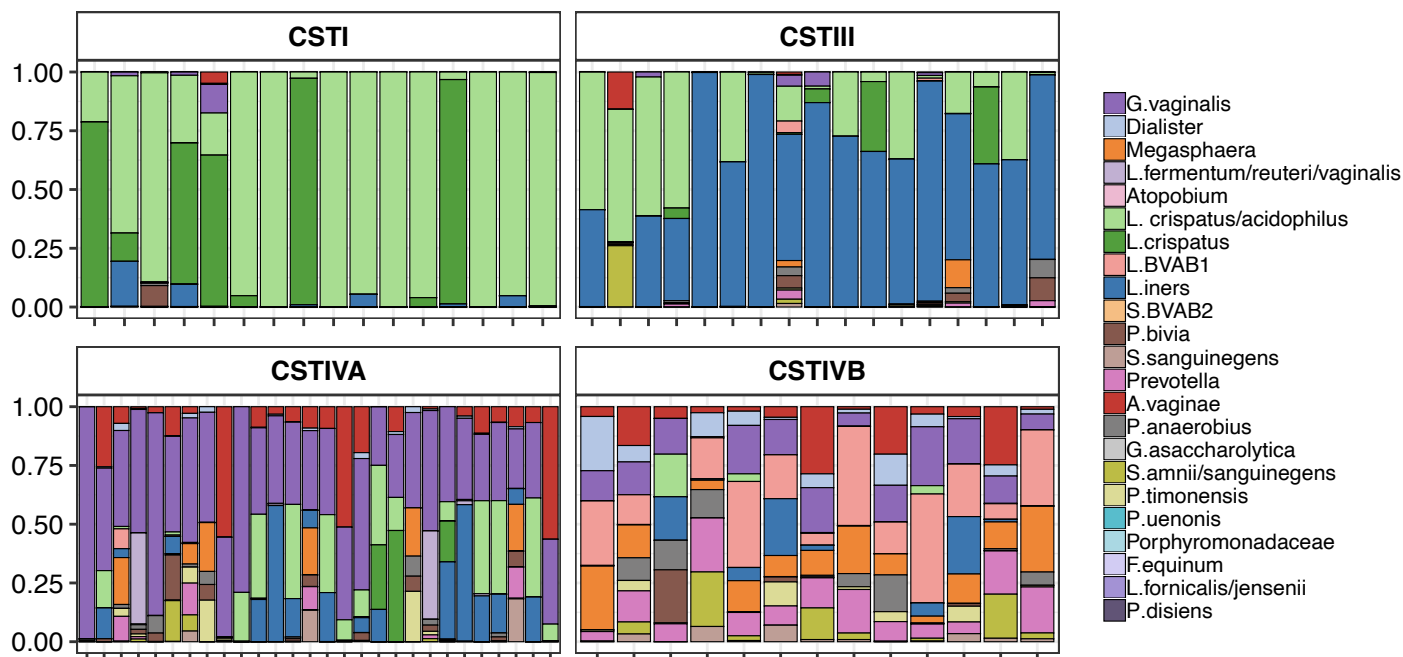

Supplement: Supplementary file 6 — Supplementary Figure S4. [file 41598_2022_13475_MOESM6_ESM.pdf]
